# Phosphoregulation of tropomyosin-actin interaction revealed using a genetic code expansion strategy

**DOI:** 10.12688/wellcomeopenres.16082.1

**Published:** 2020-07-07

**Authors:** Saravanan Palani, Darius Koester, Mohan K. Balasubramanian

**Affiliations:** 1 Centre for Mechanochemical Cell Biology, Division of Biomedical Sciences, Warwick Medical School, University of Warwick, Coventry, CV4 7AL, UK

**Keywords:** Cytokineses, Actin, Tropomyosin, Synthetic biology

## Abstract

Tropomyosins are coiled-coil proteins that regulate the stability and / or function of actin cytoskeleton in muscle and non-muscle cells through direct binding of actin filaments. Recently, using the fission yeast, we discovered a new mechanism by which phosphorylation of serine 125 of tropomyosin (Cdc8), reduced its affinity for actin filaments thereby providing access for the actin severing protein Adf1/Cofilin to actin filaments causing instability of actin filaments. Here we use a genetic code expansion strategy to directly examine this conclusion. We produced in *Escherichia coli* Cdc8-tropomyosin bearing a phosphate group on Serine-125 (Cdc8 ^PS125^), using an orthogonal tRNA-tRNA synthetase pair that directly incorporates phosphoserine into proteins in response to a UAG codon in the corresponding mRNA. We show using total internal reflection (TIRF) microscopy that, whereas *E.coli* produced Cdc8 ^PS125^ does not bind actin filaments, Cdc8 ^PS125^ incubated with lambda phosphatase binds actin filaments. This work directly demonstrates that a phosphate moiety present on serine 125 leads to decreased affinity of Cdc8-tropomyosin for actin filaments. We also extend the work to demonstrate the usefulness of the genetic code expansion approach in imaging actin cytoskeletal components.

## Introduction

The actin cytoskeleton plays a vast array of physiological roles, ranging from cell morphogenesis and cell division to developmental pattern formation and muscle contraction ( Cheffings *et al.,* 2016; Pollard & Wu, 2010; Szent-Gyorgyi, 2004). The coiled-coil protein tropomyosin is a key actin filament binding protein, which regulates actin cytoskeletal architecture and function ( Gunning *et al.,* 2015; Khaitlina, 2015; Longley, 1975). In its well-characterized role in muscle contraction, tropomyosin regulates the interaction between the motor protein myosin II and filamentous actin (F-actin), in a calcium and troponin dependent manner ( Chalovich *et al.,* 1981; Gergely, 1974; Khaitlina, 2015; Perry, 2001; Spudich & Watt, 1971; Szent-Gyorgyi, 1975). Recently, we have shown that in non-muscle cells tropomyosin function is regulated in part by phosphorylation ( Palani *et al.,* 2019). We have shown that fission yeast Cdc8-tropomyosin is phosphorylated on Serine-125 *in vivo* and that a phosphomimetic mutant protein (Cdc8S125E) shows reduced affinity for F-actin in sedimentation assays as well as in TIRF microscopy-based assays. We also showed that incubation of unphosphorylated Cdc8 with its kinase, Pom1 and adenosine triphosphate (ATP), caused release of pre-existing Cdc8-tropomyosin from actin filaments. We proposed that Cdc8-tropomyosin protected actin filaments from Adf1/cofilin mediated severing, and that phosphorylation exposed actin filaments for severing by Adf1/cofilin, thereby providing a mechanism for actin filament turnover ( Palani *et al.,* 2019). While these *in vitro* experiments (using best available current strategies) strongly pointed to phosphoregulation of Cdc8-tropomyosin interaction with actin, they still left open some caveats, since the Cdc8 proteins used in sedimentation and / or TIRF assays were not singly phosphorylated on S125, but were either phosphomimetic or present in complex mixtures containing the kinase and ATP. Here using a genetic code expansion strategy ( Neumann, 2012; Neumann-Staubitz & Neumann, 2016; Wang, 2017), we generate Cdc8 that bears a single phosphate group on Serine 125 and provide direct evidence for regulation of Cdc8-tropomyosin interaction with actin filaments via Serine 125 phosphorylation.

## Results

**Figure 1.**
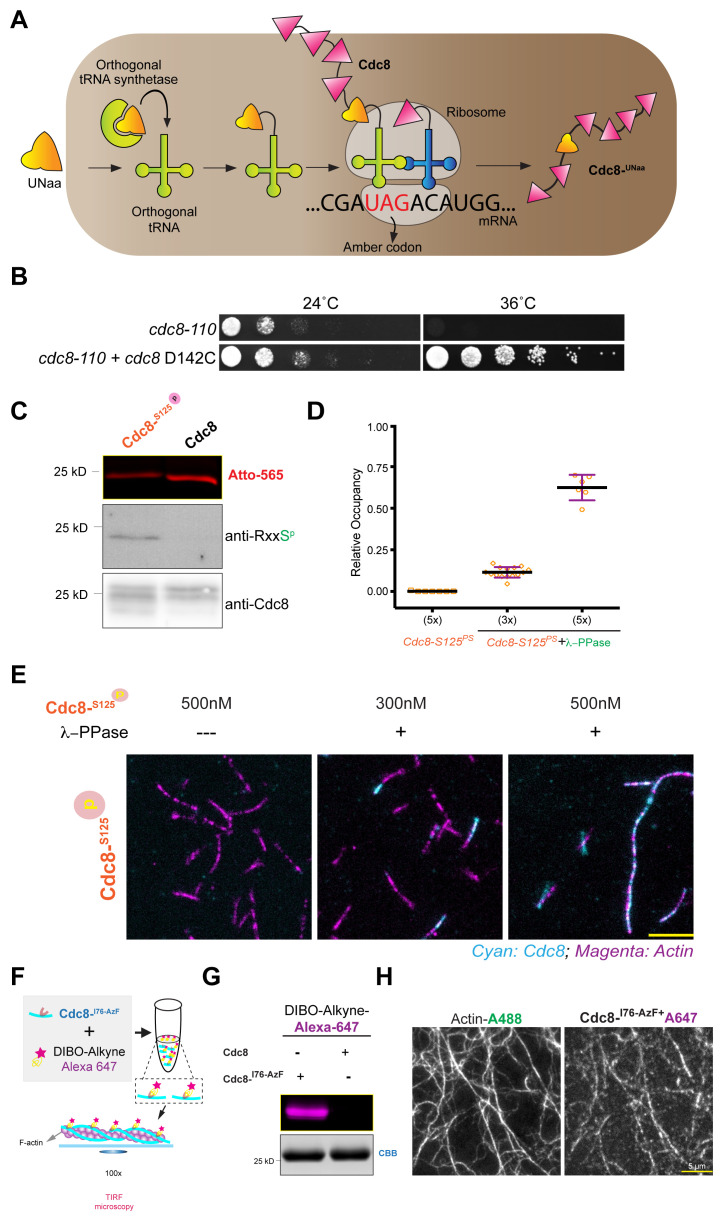
Generation of genetically encoded phosphorylated Cdc8p. **a**. Schematic representation of the production of genetically encoded Cdc8 ^PS125 ^using genetic code expansion method (SepOTS-Lambda). **b**. Ten-fold serial dilutions of cdc8+, carrying D142C in a cdc8-110 background were spotted onto YEA plates and grown for 2 days at 24°C and 36°C. **c**. Purified ASCdc8-PS125 protein was resolved on SDS-PAGE gel and blotted using antibodies against RxxSp or Cdc8 and Fluorescence gel of a purified acetylation mimicking version of Cdc8 ^PS125^-D142C labelled with atto-565. **d**. Corresponding boxplot of relative F-actin decoration by Cdc8 ^PS125^-D142C with and without lambda-phosphatase; N = [10, 15, 7] filaments. **e**. Images showing decoration of 125 nM F-actin-Alexa488 (magenta) with indicated concentrations of genetically encoded Cdc8 ^PS125^-D142C-Atto-565 (cyan) without or after 30 min incubation with λ-Phosphatase; scale bar: 5 µm; channels were merged using ImageJ (version 1.52p). **f**. Schematic representation of genetically encoded Cdc8-I76- ^AzF^ using genetic code expansion method and Azide-Alkyne click reaction with DIBO-Alkyne-Alexa dyes. **g**. Fluorescence gel of a purified acetylation mimicking version of Cdc8-I76- ^AzF^ labelled with DIBO-Alkyne-Alexa647 and Coomassie brilliant blue stained gel. **h**. Images showing decoration of 125 nM F-actin-Alexa488 with 500 nM of genetically encoded Cdc8-I76 ^AzF^ labelled with DIBO-Alkyne-Alexa647 (Cdc8 ^76AF647^); scale bar: 5 µm; channels were merged using ImageJ (version 1.52p).

To firmly establish the role of phosphorylation of serine-125 on Cdc8-tropomyosin on actin binding, we used a genetic code expansion strategy to produce Cdc8 ^PS125^ ( Figure 1A). In this strategy, we used an orthogonal tRNA-tRNA synthetase pair from *Methanocaldococcus jannaschii* (tRNA ^Cys^) and *Methanococcus maripaludis* aminoacyl tRNA synthetase for O-phosphoserine (P ^Ser^) ( Pirman *et al.,* 2015). Further, the anticodon loop in tRNA ^Cys^ was altered such that it would base-pair with the amber codon. This system for expression of P ^Ser^ bearing proteins has been pioneered by Soll and colleagues ( Park *et al.,* 2011) and further improved by Rinehart and colleagues (which we have used in this study) ( Pirman *et al.,* 2015). To produce Cdc8 bearing P ^Ser^ at position 125, we made an *E. coli* expression construct in which the codon for Serine-125 was replaced with an amber (UAG) codon. This construct was expressed in an engineered *E. coli* devoid of UAG-codons, and expressing the orthogonal tRNA ^sep^-tRNA synthetase pair for P ^Ser^ and also bearing a mutation in translational elongation factor (EF-Tu) to facilitate incorporation in response to an UAG codon ( Pirman *et al.,* 2015). We also replaced the codon for Aspartic Acid position 142 with a codon for Cysteine as described previously to facilitate fluorescent labelling of the recombinant Cdc8 ( Christensen *et al.,* 2017). We established that the presence of a Cysteine residue at position 142 did not impair Cdc8 function, since Cdc8D142C was able to rescue a *cdc8*-110 mutant for colony formation at the restrictive temperature of 36ºC ( Figure 1B ( Palani, 2020a)). Cdc8 and Cdc8 ^PS125^ expressed in *E. coli* were labelled with Atto-565 ( Figure 1C; top panel ( Palani, 2020b)). Although polyclonal antibodies against Cdc8 recognized both proteins ( Figure 1C; middle panel ( Palani, 2020b)), an antibody against phosphorylated RXXS (which we have shown previously recognizes Cdc8 ^PS125^), only detected the Cdc8 ^PS125^ produced in *E. coli* and did not recognize unphosphorylated Cdc8 produced in *E. coli* ( Figure 1C; bottom panel ( Palani, 2020b)). Note that all Cdc8-tropomyosins expressed in *E. coli* had an N-terminal acetylation mimicking sequence, as described previously ( Christensen *et al.,* 2017; Skoumpla *et al.,* 2007), due to the importance of acetylation in tropomyosin function. We then tested the ability of Cdc8 ^PS125^ to bind actin filaments in TIRF assays that we have described previously ( Palani *et al.,* 2019). Consistent with our previous experiments with Cdc8S125E, 0.3µM Cdc8 ^PS125^ failed to bind actin filaments ( Figure 1D and E ( Palani, 2020c)) ( Palani *et al.,* 2019). Importantly, treatment of Cdc8 ^PS125^ with λ-phosphatase allowed its binding to actin filaments, again consistent with previous conclusions that Cdc8-bound actin filaments more efficiently in a Serine-125 unphosphorylated state ( Figure 1D and E ( Palani, 2020c)).

Given the strength of the genetic code expansion approach, we attempted to further its use by introducing a fluorescent label on Cdc8 to facilitate its imaging by total internal reflection fluorescence microscopy (TIRFM). For this purpose, we used a tRNA-tRNA synthetase pair that introduced azido-phenylalanine (AzF) in response to an amber-UAG codon ( Chin *et al.,* 2002; Ge *et al.,* 2016). Using this orthogonal tRNA-tRNA synthetase pair, we introduced AzF into Cdc8 at position 76. Previous work has shown that substitutions introduced at this position did not affect Cdc8 function ( Christensen *et al.,* 2017; Palani *et al.,* 2019). Purified Cdc8- ^I76AzF^ was reacted with a strained-alkyne coupled to Alexa-647 in an azide-alkyne cycloaddition reaction, which generated a fluorescently labelled Cdc8 ( Figure 1F and G ( Palani, 2020d)). Cdc8 ^76AF647^ was tested for its ability to bind actin filaments using TIRFM by mixing it with F-actin. In this assay we found that Cdc8 ^76AF647^ strongly bound actin filaments ( Figure 1H ( Palani, 2020e)).

## Conclusions

In this work we have used genetic code expansion to unequivocally establish that interaction between Cdc8-tropomyosin and actin is inhibited by the presence of a phosphate group on Serine-125. These findings are consistent with our previous work revealing phosphoregulation of Cdc8-tropomyosin function ( Palani *et al.,* 2019). The recent discovery of synthetases for phosphothreonine ( Zhang *et al.,* 2017) and phosphotyrosine ( Luo *et al.,* 2017; Steinfeld *et al.,* 2014), as well as the ability to introduce three different non-canonical amino acids using three different tRNA-tRNA synthetase pairs simultaneously should facilitate further investigations of phosphoregulation of the actin cytoskeleton *in vitro*. In other work reported herein, we have fluorescently-labelled Cdc8 tropomyosin using a combination of genetic code expansion and azide-alkyne click chemistry. This method should be broadly applicable and should facilitate introduction of the fluorochrome into proteins, both for TIRFM as well as for homo-FRET experiments. In particular, this approach circumvents difficulties caused by an inability to generate single-cysteine bearing proteins, a routine approach used in protein labelling. We conclude that the expanded genetic code can be used as a powerful tool to further investigate the actin cytoskeleton and its post-translational modifications.

## Methods

### Unnatural amino acid incorporation, protein purification and labelling

For phospho-serine (pSer) incorporation: To genetically encode phosphorylated Cdc8, pGEX-ASCdc8 was used to introduce the amber codon (TAG) at position S125. Phosphorylated version of Cdc8 ^PS125^ was expressed in C321. ∆A; ∆ *serB* or BL21 ∆ *serB* cells (Addgene: 68306; 34929 ( Park *et al.,* 2011; Pirman *et al.,* 2015)); carrying pGEX-AS (acetylation mimic) Cdc8-S125TAG-D142C, SepOTS-lambda (Addgene: 68292; ( Pirman *et al.,* 2015)). Cells carrying both the plasmids were grown overnight at 30 °C at 220 rpm. Cells were diluted to 0.2 optical density (O.D) in 250 ml of Luria Bertani (LB) with antibiotics, 2 mM O-phospho-L-serine (OPLS), and protein expression was induced with 1 mM isopropyl β-D-1-thiogalactopyranoside (IPTG) at 0.6-0.8 OD and protein was expressed for 20 h at 20 °C at 220 rpm. Cells were collected and spun down at 4,000g for 20 min at 4 °C., Cells were resuspended in 50 ml lysis buffer (20 mM Tris pH 7.5, 100 mM NaCl, 2 mM EGTA, 5 mM MgCl _2_, 50 mM NaF, 1 mM NaVO4 and 1x PhosSTOP, Roche), lysed by sonication and heated to 90ºC. Insoluble components were removed by centrifugation at 21000g for 30min at 4 °C and the resulting supernatant was incubated with 10 mg/l DNase and 10 mg/l RNase at 4ºC for 1 hr. Soluble Cdc8 was precipitated at pH4.5 and resuspended in 5 mM Tris-HCl pH 7.0 and further dialyzed overnight with storage buffer (20 mM Tris-HCl, pH 7.5, 50 mM NaCl, 0.01% NaN3, 1 mM DTT). ( Palani *et al.,* 2019; Skau *et al.,* 2009; Skoumpla *et al.,* 2007). Phosphorylated Cdc8 was labelled at single cysteine residue either at D142C, by atto-565 maleimide (AD 565-41, ATTO-TEC, GmbH).

### Western blotting

Phosphorylation of Cdc8 at position S125 was confirmed by immunoblot. Purified tropomyosin was resolved in 12% SDS-PAGE gel electrophoresis system (Biorad USA). Blotting was done using PVDF membrane after transfer, it was incubated with 5% bovine serum albumin (BSA) overnight. Primary antibodies (monoclonal rabbit anti-RxxS at 1:200, [(110B7E) Rabbit mAb #9614, Cell Signalling, USA] and polyclonal rabbit anti-Cdc8 [Gift from Sarah-Hitchcock Degrogi, USA, at 1:1000) were prepared in 5% bovine serum albumin (BSA) and incubated overnight at 4°C with shaking. Immunoblots were developed using polyclonal goat anti rabbit (ab6702, Abcam, UK) peroxidase conjugated secondary antibodies (1:3000) in combination with chemiluminescent HRP substrate (Clarity western ECL, #1705060, BIO-RAD, USA) and developed using ChemidocMP (Biorad).

For Azido Phenylalanine (AzF) incorporation: To genetically encode Azidophenylalanine into Cdc8 at position I76, pET-ASCdc8 was used to introduce the amber codon (TAG) at position I76. AzF incorporated version of Cdc8-I76 ^-AzF^ was expressed in BL21-Ai ; carrying pET-AS (acetylation mimic) Cdc8- Cdc8-I76TAG, pEVOL-pAzF (Addgene: 31186) or pDULE2-CNF (Addgene: 85495 ( Miyake-Stoner *et al.,* 2009) in the presence of 2mM Azido-phenylalanine (Bachem, cat no: 4096192) for 12h at 37°C as described ( Peeler & Mehl, 2012). Azido-phenylalanine incorporated Cdc8 was purified as described in the previous section. Purified Cdc8-I76 ^-AzF^ was incubated with Alkyne-Alexa647 (Click-iT™ Alexa Fluor™ 647 sDIBO Alkyne, cat no: C20022, Thermo-Fisher) (1:20 ratio) at 16°C for 12-16 hr.

Actin was purified from rabbit skeletal muscle acetone powder as described previously ( Spudich & Watt, 1971). Actin was labeled with maleimide-Alexa488 (Molecular Probes).

### Tropomyosin coating of actin filaments-TIRFM

TIRFM experiments to visualize tropomyosin coated actin filaments were prepared and conducted following established protocols ( Palani *et al.,* 2019). Glass coverslips (#1.5 boroslicate, Menzel, Germany, Fisher Scientific cat. No.: 11348503) were cleaned with Hellmanex III (Hellma Analytics, Mühlheim, Germany, Merck cat. No.: Z805939) following the manufacturer's instructions, rinsed thoroughly with MilliQ water and blow dried with N _2 _gas. Experimental chambers were assembled by sticking the middle section of 0.2 ml PCR tubes (Starlab, UK, cat. no.: SLI1402-3708) (without the lid and the conical bottom) to the cleaned glass using UV glue (NOA68, Norland Products, Cranbury, NJ) by curing for three minutes in intense UV light at 265 nm (UV Stratalinker 2400, Stratagene, USA). Passivation of the surface of freshly cleaned and assembled chambers was performed by incubation with 1mg/ml PLL-PEG(2 kDa) (SUSOS AG, Switzerland, cat. no.: PLL(20)-g[3.5]-PEG(2)) (for ASCdc8 loading curves) for 20min followed by three washes with KMEH (50mM KCl, 2 mM MgCl _2_, 1 mM EGTA, 20 mM HEPES, pH 7.2).

Polymerization of F-actin was achieved by a 30 min incubation of 5 µM G-actin (purified from aceton powder, Merck, cat. no.: M6890-10G) at a labelling ratio of 5% _mol_ (Alexa488-labelled G-actin) in a final buffer at 50mM KCl, 2 mM MgCl _2_, 1 mM EGTA, 20 mM HEPES, pH 7.2 supplemented with 2mM Mg-ATP as described earlier ( Palani *et al.,* 2019). Next, F-actin was diluted to a concentration of 1.25 µM in KMEH supplemented with 0.5% Methylcellulose (Fisher Scientific, cat. no.: 11480041) and incubated for 10 min with 3 or 5 µM labeled ASCdc8 variants or 5 µM DIBO-Alkyne-Alexa-647 labeled Cdc8-I76-AzF (Cdc8 ^76AF647^) in aliquots at a final volume of 10µl. The content of each aliquot was added to a separate experimental chamber filled with 90µl of KMEH supplemented with 0.5% Methylcellulose. 

In experiments using λ-Phosphatase, 200nM λ-Phosphatase (Lambda PP, New England BioLabs, USA, cat. no.: P0753S) were incubated together with 1.25 µM F-actin and 3 or 5 µM Cdc8 ^PS125^-D142C labelled with atto-565 for 30min before addition to the experimental chambers.

Images were acquired using a Nikon Eclipse Ti-E/B microscope equipped with perfect focus system, a Ti-E TIRF illuminator (CW laser lines: 488nm, 561nm and 640nm) and a Zyla sCMOS 4.2 camera (Andor, Oxford Instruments, UK) controlled by Andor iQ3 software.

### Quantification of F-actin decoration by Cdc8 ^PS125^-D142C

For the generation of the actin loading graphs (Fig. 1D), the Cdc8PS125-D142C decoration length and F-actin length were measured manually for each filament in ImageJ (version 1.52p; NIH, USA) and their ratio was computed and plotted.

### Statistical analysis

Data was plotted as box plots depicting individual data points, the mean values (black lines) and standard deviation (whiskers) using Graph pad Prism version 6.

## Data availability

### Underlying data

Figshare: Figure 1B. https://doi.org/10.6084/m9.figshare.12490022.v1 ( Palani, 2020a)

This project contains the following underlying data:

- Spot test on a YEA plate scanned image (Unedited scanned YEA plate in .tif format)

Figshare: Figure 1C. https://doi.org/10.6084/m9.figshare.12490031.v1 ( Palani, 2020b)

This project contains the following underlying data:

- PhosphoCdc8 western blots and maleimide labelled fluorescent gel of phosphoCdc8 (Unedited western blot scan and fluorescent SDS-AGE gel in .tif format)

Figshare: Figure 1E. https://doi.org/10.6084/m9.figshare.12490058.v1 ( Palani, 2020c)

This project contains the following underlying data:

- TIRFM images of phospho-tropomyosin loading onto actin filaments with and without phosphatase (Uncropped TIRF images in .tif format)

Figshare: Figure 1G. https://doi.org/10.6084/m9.figshare.12490067.v1 ( Palani, 2020d)

This project contains the following underlying data:

- Azide-alkyne click labelled fluorescent and coomassie stained gels of tropomyosin (Uncropped fluorescent SDS-AGE and Coomassie stained gel in .tif format)

Figshare: Figure 1H. https://doi.org/10.6084/m9.figshare.12490115.v1 ( Palani, 2020e)

This project contains the following underlying data:

- TIRFM images of azide-alkyne click labelled Cdc8 loading onto actin filaments (Uncropped TIRF images in .tif format)
