# Supplementary material for: Phosphoregulation of tropomyosin-actin interaction revealed using a genetic code expansion strategy
Source: Wellcome Open Res. 2020 Jul 7;5:161. [Version 1] doi: 10.12688/wellcomeopenres.16082.1 (PMC7411518; doi:10.12688/wellcomeopenres.16082.1)
Supplement: Peer review history

# Peer review: Reviewer response for version 1

**DOI:** 10.21956/wellcomeopenres.17646.r39678

**Published:** 2020-08-06

**Authors:** Gislene Pereira

**Affiliations:** 1 Centre for Organismal Studies, Centre for Molecular and Cell Biology, University of Heidelberg, German Cancer Research Centre, Heidelberg, Germany

In this manuscript, Palani *et al*. have described the method and use of genetic code expansion (GCE) by using non-canonical amino acids (ncAA’s). The authors have successfully incorporated phospho-serine (pSER) and Azido-phenylalanine (AzF) into tropomyosin. This method paper is a follow up of their previous work on tropomyosin regulation by post-translational modification and binding to actin. This work now clearly shows that ncAA’s can be used for the functional analysis of the role of phosphorylation instead of conventional phospho-mimetic substitutions (D or E). In particular, labeling strategy using click chemistry-based method (Azide-Alkyne) is a very interesting approach. It will be highly appreciated in the actomyosin field, where there are a lot of artifacts in labeling cytoskeleton proteins by conventional labeling strategies.

 Overall, the key findings in the paper are interesting and very well supported by the experiments and are in line with their previously published work. In my opinion, the use of unnatural amino acid technology to study post-translational modification and labeling of actin cytoskeleton proteins is very appealing. It has the potential of opening up new avenues in other fields of research, being of general interest.

 The authors should address the following minor points prior to indexing. 

1. The authors should discuss the possibilities of incorporating more than one ncAA’s (for example pSER) or pSER in addition to other ncAA’s into proteins.
2. Please explain the 3x and 5x (Figure 1D) clearly in the figure legend or methods section. 
3. Authors should comment on using a non-hydrolysable phosphonate analog (DL-AP4) incorporation method developed by Chin’s lab (Rogerson *et al*., 2015) ^1^. 
4. Please include a few sentences about using GCE strategy to incorporate pSER in mammalian cells and if this could be used to study the function of proteins *in vivo*. Please cite Beranek *et al*., 2018 ^2^.

Is the work clearly and accurately presented and does it cite the current literature?

Yes

If applicable, is the statistical analysis and its interpretation appropriate?

Yes

Are all the source data underlying the results available to ensure full reproducibility?

Yes

Is the study design appropriate and is the work technically sound?

Yes

Are the conclusions drawn adequately supported by the results?

Yes

Are sufficient details of methods and analysis provided to allow replication by others?

Yes

Reviewer Expertise:

Cell cycle, cytokinesis, mitotic checkpoint, ciliogenesis.

I confirm that I have read this submission and believe that I have an appropriate level of expertise to confirm that it is of an acceptable scientific standard.

## References

[1] : Efficient genetic encoding of phosphoserine and its nonhydrolyzable analog. *Nat Chem Biol*.2015;11(7) : 10.1038/nchembio.1823 496-503 10.1038/nchembio.1823 26030730PMC4830402

[2] : Genetically Encoded Protein Phosphorylation in Mammalian Cells. *Cell Chemical Biology*.2018;25(9) : 10.1016/j.chembiol.2018.05.013 1067-1074.e5 10.1016/j.chembiol.2018.05.013 PMC616234529937407

# Peer review: Reviewer response for version 1

**DOI:** 10.21956/wellcomeopenres.17646.r39435

**Published:** 2020-07-20

**Authors:** Dannel McCollum

**Affiliations:** 1 Department of Biochemistry & Molecular Pharmacology, University of Massachusetts Medical School, Worcester, MA, USA

This report describes the use of unnatural amino acid substitutions to investigate biochemical properties of the actin cytoskeletal protein tropomyosin (Cdc8). In particular, they show that they can incorporate azido-phenylalanine to fluorescently label Cdc8 tropomyosin and phospho-serine to assess the effect of phosphorylation of a particular serine residue. This work follows up on earlier work from the same lab showing that phosphorylation of serine-125 inhibits the ability of Cdc8 to bind actin filaments. While the evidence from the previous paper was very strong, for various reasons it is difficult to 100% prove that observed effects are from phosphorylation of a particular amino acid. For example, phospho-mimetic substitutions only partially mimic phosphorylation, and phosphorylating a protein *in vitro* typically results in additional sites being phosphorylated. This work clearly shows that serine-125 phosphorylation of Cdc8 is sufficient to inhibit its actin binding. Phosphorylation plays an important role in regulation of the actin cytoskeleton, and this study shows the feasibility of this sort of approach for investigating the role of phosphorylation in regulation of the actin cytoskeleton. I have a couple minor comments that the authors might want to consider.

 Minor Comments.

1. In principle, proteins incorporating unnatural phosphorylated amino acids in *E. coli* could be dephosphorylated. Even with low rates of dephosphorylation, most of the protein would be quickly dephosphorylated unless it is being rephosphorylated or rapidly turned over. Their data suggest that this might not be a major concern, but perhaps the authors could comment on this issue. 
2. In Figure 1D, it is a bit confusing what “(5x)” and “(3x)” refer to. This is not explained in the legend. It also seems like 1E should precede 1D since 1D is showing quantification of what is shown in 1E.

Is the work clearly and accurately presented and does it cite the current literature?

Yes

If applicable, is the statistical analysis and its interpretation appropriate?

Yes

Are all the source data underlying the results available to ensure full reproducibility?

Yes

Is the study design appropriate and is the work technically sound?

Yes

Are the conclusions drawn adequately supported by the results?

Yes

Are sufficient details of methods and analysis provided to allow replication by others?

Yes

Reviewer Expertise:

Protein phosphorylation. Regulation of the cytoskeleton.

I confirm that I have read this submission and believe that I have an appropriate level of expertise to confirm that it is of an acceptable scientific standard.
